# Association of Serum Hepatocyte Growth Factor Level with Systemic Inflammatory Biomarkers in Patients with Pancreatobiliary Cancer

**DOI:** 10.5152/tjg.2023.22124

**Published:** 2023-05-01

**Authors:** İlkay Tuğba Ünek, İlhan Öztop, Yasemin Başbınar, Tarkan Ünek, Asım Leblebici, Ece Çakıroğlu, Tuğba Uysal, Baran Akagündüz, Hülya Ellidokuz, İbrahim Astarcıoğlu

**Affiliations:** 1 Department of Medical Oncology, Dokuz Eylül University Faculty of Medicine, İzmir, Turkey; 2 Department of Translational Oncology, Dokuz Eylül University Institute of Oncology, İzmir, Turkey; 3 Department of General Surgery, Dokuz Eylül University Faculty of Medicine, İzmir, Turkey; 4 Department of Translational Oncology, Dokuz Eylül University Institute of Health Sciences, İzmir, Turkey; 5 Department of Genomics and Molecular Biotechnology, Dokuz Eylül University, İzmir International Biomedicine and Genome Institute, İzmir, Turkey; 6 Department of Molecular Biology and Genetics, Hitit University Faculty of Science and Art, Çorum, Turkey; 7 Department of Medical Oncology, Erzincan Binali Yıldırım University Faculty of Medicine, Erzincan, Turkey; 8 Department of Preventive Oncology, Dokuz Eylül University Institute of Oncology, İzmir, Turkey

**Keywords:** Hepatocyte growth factor, inflammation, pancreatobiliary cancer

## Abstract

**Background:**

Hepatocyte growth factor is a cytokine secreted by the stromal cells in the tumor microenvironment. There is little information about the clinical significance of serum hepatocyte growth factor level in patients diagnosed with pancreatobiliary cancer. The objective of the current study was to investigate the relationship between serum hepatocyte growth factor level with inflammation markers and the clinical features of patients with pancreatobiliary cancer.

**Methods:**

A total of 62 patients with pancreatobiliary cancer were included in this study. Serum hepatocyte growth factor concentrations were evaluated utilizing the enzyme-linked immunosorbent assay method.

**Results:**

The median serum hepatocyte growth factor level was 329.1 ng/mL (1.4-1051.1). The patients were categorized into 2 groups as those below the median hepatocyte growth factor level (low hepatocyte growth factor) and those above the median hepatocyte growth factor level (high hepatocyte growth factor). While 40.9% of the patients without metastasis were observed to be in the high hepatocyte growth factor group, 72.2% of the metastatic patients were observed to be in the high hepatocyte growth factor group (*P* = .025). The median levels of monocyte, monocyte-to-lymphocyte ratio, C-reactive protein, and C-reactive protein-to-albumin ratio were found to be significantly higher in the high hepatocyte growth factor group as compared to the low hepatocyte growth factor group (*P* < .050).

**Conclusion:**

The significant relationship between serum hepatocyte growth factor level and systemic inflammation markers in patients with pancreatobiliary cancer is shown for the first time in our study. This study, which showed a significant relationship between the presence of metastasis and serum hepatocyte growth factor level, suggests that serum hepatocyte growth factor level may be a prognostic biomarker in patients who are diagnosed with pancreatobiliary cancer.

**Main Points**

- In our study, the presence of statistically significantly higher median levels of monocyte, monocyte-to-lymphocyte ratio, C-reactive protein, and C-reactive protein-to-albumin ratio in the high hepatocyte growth factor (HGF) group as compared to the low HGF group shows the significant relationship between the serum HGF level and inflammation in pancreatobiliary cancers.
- The significant relationship between the serum HGF level and systemic inflammation markers in patients with pancreatobiliary cancer is shown for the first time in our study.
- While 40.9% of the patients without metastasis were found in the high HGF group, 72.2% of the metastatic patients were found in the high HGF group (*P* = .025).
- Our study, which found a statistically significant relationship between the serum HGF levels and the presence of metastasis in patients with pancreatobiliary cancer, draws attention to the critical role played by the HGF/c-MET signaling pathway in the development of metastasis and suggests that serum HGF level may be a prognostic biomarker in patients with pancreatobiliary cancer.

## Introduction

Pancreatobiliary cancers are very aggressive and resistant to antineoplastic treatments. Pancreatic cancer is among the top deadly cancer types as it is the fourth leading cause of cancer death, while biliary tract cancer is the fifth and seventh leading cause of cancer death in males and females, respectively. Since the majority of patients with pancreatobiliary cancer are either locally advanced or metastatic at the time of presentation, they are ineligible for surgical resection. Operated patients develop high rates of recurrence in the early postoperative period. Diagnostic, prognostic, and predictive biomarker discovery is crucial in improving the poor prognosis of patients with pancreatobiliary cancer. Despite all the efforts so far, accurate diagnostic, prognostic, and predictive biomarkers for pancreatobiliary cancers have not been determined.^1-3^

Hepatocyte growth factor (HGF) is a cytokine secreted by stromal cells in the tumor microenvironment and binds to “mesenchymal-epithelial transition (c-MET) factor,” which is a proto-oncogenic receptor and activates the HGF/c-MET signaling pathway. The HGF/c-MET signaling pathway plays an important role in embryonic development and wound healing. The HGF/c-MET signaling pathway is rarely activated in adults except in cancer. The overproduction of HGF, *MET* mutation, *MET* amplification, and c-MET protein overexpression causes aberrant activation of the HGF/c-MET signaling pathway. The hyperactivity of the HGF/c-MET signaling pathway stimulates cell survival, proliferation, migration, invasion, angiogenesis, and metastasis.^4,5^

The overexpression of c-MET protein has been demonstrated in tumor tissue in many types of cancer and it is associated with poor prognosis.^6-9^ Although there are studies indicating that c-MET overexpression in tumor tissue of patients with pancreatobiliary cancer is a prognostic biomarker, there is little information about the clinical significance of the blood HGF level of the patients. Conflicting results have been obtained in studies investigating the diagnostic value of blood HGF level of patients with pancreatobiliary cancer.^10-12^ In a study evaluating the prognostic importance of the blood HGF level, it was determined that the blood HGF level in patients having pancreatic cancer was observed to have significantly higher levels in the advanced tumor stage, in the presence of lymph nodes and distant metastases, and it was stated that the blood HGF level could reflect the severity of the cancer.^12^

There is a close relationship between inflammation and cancer.^13^ Increased levels of peripheral blood leukocytes, neutrophils, monocytes, neutrophil-to-lymphocyte ratio (NLR), monocyte-to-lymphocyte ratio (MLR), platelet-to-lymphocyte ratio (PLR), C-reactive protein (CRP), CRP-to-albumin ratio (CAR), and systemic immune-inflammation (SII) index have been associated with poor prognosis in patients with pancreatobiliary cancer, and the importance of inflammation in cancer biology has been emphasized.^14-20^

The inflammatory cells in the tumor microenvironment generally create an immunosuppressive environment. There is increasing evidence which shows that cancer-associated inflammation has a critical part in tumor progression.^13^ A relationship has been demonstrated between the inflammation and activation of the HGF/c-MET signaling pathway in a tumor microenvironment. Inflammatory mediators in the tumor microenvironment increase the HGF gene expression in stromal cells.^21^ It was determined that the expression of HGF and c-MET in the tissue increased significantly during the development of experimental chronic pancreatitis.^22^ Studies detecting high HGF levels in the blood in acute and chronic pancreatitis after hepatopancreatic surgery are among the studies evaluating the relationship of HGF with inflammation.^23-25^

After a literature review, it is seen that there are no studies assessing the association of serum HGF level with systemic inflammation markers in patients with pancreatobiliary cancer. In the current study, the objective was to investigate the relationship between the serum HGF level with inflammation markers and clinical features of patients with pancreatobiliary cancer.

## MATERIALS AND METHODS

### Patients

In this study, there was a total of 69 patients included, who were scheduled for an operation or systemic treatment due to a diagnosis of biliary tract, pancreatic or ampullary carcinoma at Dokuz Eylul University, Faculty of Medicine, Departments of General Surgery and Medical Oncology. We obtained the informed consent of the patients and the approval of the local ethical committee for this study before commencing the study (date: June 26, 2014; decision no: 2014/23-21). Among the operated patients, 7 patients who were not found to have adenocarcinoma in the pathological examination of the operation material [1 patient with intraductal papillary mucinous neoplasia, 2 patients with solid pseudopapillary tumor, 1 patient with neuroendocrine tumor, 1 patient with neuroendocrine carcinoma, 1 patient with choledocholithiasis, and 1 patient with chronic pancreatitis /cholangitis] were excluded from the study. The data on clinical characteristics, including demographic data, tumor type, stage, carbohydrate antigen (Ca)19-9, complete blood counts, albumin, and CRP were collected. All the laboratory parameters were assayed during routine workups before the treatment. Neutrophil-to-lymphocyte ratio was defined as the ratio of the absolute neutrophil count to the absolute lymphocyte count. Similarly, MLR and PLR were defined as the absolute monocyte count divided by the absolute lymphocyte count, and the absolute platelet count divided by the absolute lymphocyte count, respectively. C-reactive protein-to-albumin ratio was calculated by dividing the serum CRP by the albumin. Systemic immune-inflammation index was calculated by multiplying the absolute platelet and neutrophil counts and dividing by the absolute lymphocyte count.

### Blood Sample Collection and Measurement of Hepatocyte Growth Factor in Serum

Patients with operation planned had peripheral blood samples taken before their operation, while metastatic patients with systemic chemotherapy planned had their peripheral blood samples taken before chemotherapy. For HGF analysis, 8 cc of peripheral blood was placed in a serum separator tube. The collected blood samples were centrifuged at 3000 g for 15-20 minutes without any delay. Subsequently, the serum samples were stored at −20°C until analysis.

Serum HGF concentrations were evaluated by the solid-phase sandwich enzyme-linked immunosorbent assay (ELISA) method using “Sigma-Aldrich Human HGF ELISA Kit” as per the instructions manual of the manufacturer. The obtained serum samples and the standards were added to the wells, which were pre-coated with human HGF monoclonal antibody. Any HGF present was bound by the immobilized antibody. Any unbound material was washed away. The sandwich method was created by adding the second (detector) antibody. After removing the unbound antibody-enzyme reagent by the process of washing, a substrate solution was added to the wells. It was observed that the solution color turned blue, and its density was measured to be proportional to the amount of HGF in the sample. Color change was detected due to the effect of the stopping reagent and the intensity of the color was measured using an automated ELISA microplate reader. Hepatocyte growth factor results were expressed as pg/mL.

### Statistical Analysis

Analysis of the data was completed with the Statistical Package for the Social Sciences 24.0 (IBM Corp.; Armonk, NY, USA) program. Categorical variables were analyzed with chi square analysis; while the quantitative variables were analyzed with nonparametric Mann-Whitney *U* test and Kruskal–Wallis test. Pearson correlation analysis was performed. Kaplan–Meier–Log Rank (Mantel-Cox) analysis was used for survival analysis. *P <* .05 was accepted as statistically significant.

## Results

The clinical characteristics of the patients are shown in Table 1. The median HGF level was 329.1 ng/mL (1.4-1051.1) in the preoperative blood samples of 44 (71.0%) patients who underwent surgical resection as well as in the blood samples taken before chemotherapy from 18 patients (29.0%), who had metastatic disease. The patients were categorized into 2 groups as those below the median HGF level (low HGF) and those above the median HGF level (high HGF), clinical characteristics were compared between these 2 groups (Table 2). While 33.3% of the patients with pancreatic cancer were found in the high HGF group, 46.2% of the patients with ampullary cancer were found in the high HGF group, and 72.7% of the patients with biliary tract cancer were in the high HGF group (*P* = .022). While 40.9% of the patients without metastasis were found in the high HGF group, 72.2% of the metastatic patients were found in the high HGF group (*P* = .025). While the median levels of monocyte, MLR, CRP, and CAR were significantly higher in the high HGF group as compared to the low HGF group (*P* < .050); the median levels of NLR and SII were found to be higher, close to statistical significance (*P* = .050, *P* = .089, respectively).

The laboratory parameters of the patients were grouped as those below the median values (low group) and those above the median values (high group), and HGF levels were compared between these groups (Table 3). The median HGF levels were found to be statistically significantly higher in the high monocyte group, high platelet group, high SII group, high CRP group, and in the high CAR group (*P* < .050). In the high NLR group and high PLR group, the median HGF levels were found to be higher, close to statistical significance (*P* = .063, *P* = .055, respectively).

The median follow-up was 13.0 months (range: 0.17-58.13), and 51 of the 62 (82.3%) patients died. The median overall survival of the patients was found to be 12.8 months (95% CI = 7.882-17.785). The median overall survival was significantly shorter in those >65 years of age, metastatic patients, the high Ca19-9 group, and the high MLR group (*P* < .05). There was no difference in the overall survival between the low HGF group and the high HGF group (Table 4). In the multivariate cox regression analysis, age (OR = 2.146; 95% CI = 1.188-3.876, *P* = .011), presence of metastasis (OR = 2.260; 95% CI = 1.189-4.297, *P* = .013), and Ca19-9 level (OR = 2.160; 95% CI = 1.174-3.973, *P* = .013) were found to be independent predictors of survival.

## Discussion

In the current study, a significant correlation was found between the presence of metastasis and high serum HGF levels in patients with pancreatobiliary cancer (Table 2).

The HGF/c-MET signaling pathway has a critical role during the development of metastatic disease, starting from cellular dissociation within the primary tumor to cellular re-association within the metastatic niche.^7^ The binding of HGF to the c-MET receptor provides the activation of mitogen-activated protein kinase/ERK, phosphoinositide 3-kinase/Akt, signal transducer and activator of transcription-3, and focal adhesion kinase signaling pathways, which play a role in the cell survival, proliferation, migration, invasion, and metastasis.^5^ The activation of the HGF/c-MET signaling pathway increases the HGF production from cancer-associated fibroblasts in the tumor microenvironment. Hepatocyte growth factor directly stimulates angiogenesis by binding to its receptors on endothelial cells. Besides, HGF indirectly contributes to angiogenesis by stimulating other stromal cells in the tumor microenvironment to produce pro-angiogenic factors such as vascular endothelial growth factor (VEGF).^7^ In a study which examined patients diagnosed with pancreatic cancer, it was determined that those with metastasis had higher HGF levels in the blood compared to those without metastasis, but the difference was not found to be significant.^10^ Our study, which found a statistically significant relationship between the serum HGF levels and the presence of metastasis in patients with pancreatobiliary cancer, draws attention to the critical role played by the HGF/c-MET signaling pathway in the development of metastasis.

Cancer-associated inflammation components in the tumor microenvironment are myeloid cells (macrophages and neutrophils), basophils, and eosinophils. Inflammatory cells, particularly tumor-associated macrophages, promote tissue invasion, intravasation, and metastasis of tumor cells.^13^ Tumor cells often produce a variety of inflammatory chemokines, such as neutrophil-attracting CXC-chemokines. The migration of neutrophils toward the tumor is mediated by these chemokines secreted by the tumor.^26^ It has been shown that neutrophils can secrete numerous chemokines, cytokines, and angiogenic factors, such as transforming growth factor-β (TGF-β), VEGF, and HGF. Neutrophils in the tumor microenvironment play a role in tumor growth, invasion, angiogenesis, and in metastasis.^13,26,27^

There is growing evidence that the HGF/c-MET signaling pathway is involved in the regulation of neutrophil function, monocyte/macrophage differentiation, activation, and also thrombopoiesis.^28^

Besides their active roles in hemostasis and thrombosis, platelets also play a role in inflammation, tumor growth, and metastasis. Platelets promote tumor growth by secreting various growth factors such as VEGF, TGF-β, platelet-derived growth factor, and epidermal growth factor. The formation of platelet-tumor cell aggregates in the bloodstream protects tumor cells from immune system attack and facilitates their metastasis.^13,29^ In a recent study, it was shown that cancer-related thrombocytosis reduces immunity by suppressing the T lymphocyte response against the tumor.^30^

In our study, the presence of statistically significantly higher median levels of monocyte, MLR, CRP, and CAR in the high HGF group as compared to the low HGF group shows the significant relationship between the serum HGF level and inflammation in pancreatobiliary cancers (Table 2). Similarly, the presence of statistically significantly higher median values of serum HGF in high monocyte group, high platelet group, high SII group, high CRP group, and high CAR group also draws attention to the significant relationship between serum HGF level and inflammation in pancreatobiliary cancers (Table 3). The limitation of our study is the small number of patients included in the study. Therefore, it will be necessary to support our results with further studies that involve larger number of patients with pancreatobiliary cancer evaluating the relationship between serum HGF level and inflammation.

The signaling pathway of HGF/c-MET has been investigated in many solid cancer types, as it is considered an important target in the diagnosis, prognosis, and treatment of cancer. The treatments targeting the HGF/c-MET signaling pathways include MET-specific tyrosine kinase inhibitors, MET monoclonal antibodies, and HGF monoclonal antibodies.^4,5,21^ There is currently no approved therapy targeting the HGF/c-MET signaling pathway in pancreatobiliary cancers; however, clinical studies are ongoing and the signaling pathway of HGF/c-MET remains important as a therapeutic target.^6^

As a conclusion to this study, it can be stated that the significant relationship between the serum HGF level and systemic inflammation markers in this study is thought to be the reflection of the relationship between HGF and inflammation in the tumor microenvironment to the peripheral blood. The association between the serum HGF level and systemic inflammation markers in patients with pancreatobiliary cancer is shown for the first time in our study. This study, which showed a significant relationship between the presence of metastasis and serum HGF level, reveals the importance of the HGF/c-MET signaling pathway in the development of metastasis and suggests that serum HGF level may be a prognostic biomarker in patients with pancreatobiliary cancer. The serum HGF level can indicate the severity of the disease as well as serve as a guide in terms of personalized targeted treatments. Studies with larger number of patients evaluating the prognostic and predictive significance of serum HGF levels in pancreatobiliary cancers are needed.

## Figures and Tables

**Table 1.** The Clinical Characteristics of the Patients

Characteristics	Median or n (%)	Range
Age	64.0	43.0-89.0
Gender		
Male	37 (59.7)	
Female	25 (40.3)	
Tumor type		
Pancreatic cancer	27 (43.6)	
Biliary tract cancer	22 (35.5)	
Ampullary cancer	13 (20.9)	
Metastasis		
M0	44 (71.0)	
M1	18 (29.0)	
Ca 19-9 (U/mL)	62.4	0.8-38 580.0
Neu (/μL)	4900	1900-13 100
Lymph (/μL)	2100	700-4900
Monocyte (/μL)	700	200-2600
Platelet (/mL)	259 000	108 000-486 000
NLR	2.5	0.7-12.6
MLR	0.32	0.12-1.86
PLR	128.9	56.0-357.8
SII (Platelet*Neu/Lymph)	613 333.3	154 285.7-4042 888.9
Alb (g/dL)	3.6	2.3-4.8
CRP (mg/L)	9.9	0.5-233.0
CAR (CRP/Alb)	2.8	0.1-90.4
HGF (ng/mL)	329.1	1.4-1051.1

Alb, albumin; Ca 19-9, carbohydrate antigen 19-9; CAR, CRP-to-albumin ratio; CRP, C-reactive protein; HGF, hepatocyte growth factor; Lymph, lymphocyte; MLR, monocyte-to-lymphocyte ratio; Neu, neutrophil; NLR, neutrophil-to-lymphocyte ratio; PLR, platelet-to-lymphocyte ratio; SII, systemic immune-inflammation index.

**Table 2.** The Clinical Characteristics of the Patients According to HGF Groups

Characteristics	HGF ≤ 329.1 ng/mL		HGF > 329.1 ng/mL		*P*
	Median or n (%)	Range	Median or n (%)	Range	
Age	64.0	43.0-88.0	64.0	44.0-89.0	.994
Gender					.796
Male	19 (51.4)		18 (48.6)		
Female	12 (48.0)		13 (52.0)		
Tumor type					**.022**
Pancreatic cancer	18 (66.7)		9 (33.3)		
Biliary tract cancer	6 (27.3)		16 (72.7)		
Ampullary cancer	7 (53.8)		6 (46.2)		
Metastasis					**.025**
M0	26 (59.1)		18 (40.9)		
M1	5 (27.8)		13 (72.2)		
Ca 19-9 (U/mL)	40.25	0.8-6856.0	80.0	0.8-38 580.0	.413
Neu (/μL)	4550	1900-9600	5000	2900-13 100	.127
Lymph (/μL)	2200	700-4500	2000	700-4900	.268
Monocyte (/μL)	600	200-1200	800	400-2600	**.036**
Platelet (/mL)	240 500	135 000-455 000	295 000	108 000-486 000	.334
NLR	2.27	0.78-8.00	3.15	0.73-12.56	.050
MLR	0.29	0.15-1.14	0.37	0.12-1.86	**.018**
PLR	117.54	56.00-280.00	150.00	60.45-357.78	.163
SII (platelet * Neu/Lymph)	573 414.03	154 285.71-1961 692.31	727 636.36	217 636.36-4042 888.89	.089
Alb (g/dL)	3.76	2.89-4.76	3.38	2.32-4.23	**.028**
CRP (mg/L)	5.50	0.5-35.1	21.20	0.9-233.0	**.000**
CAR (CRP/Alb)	1.38	0.13-10.87	6.72	0.24-90.40	**.000**

Alb, albumin; Ca 19-9, carbohydrate antigen 19-9; CAR, CRP-to-albumin ratio; CRP, C-reactive protein; HGF, hepatocyte growth factor; Lymph, lymphocyte; MLR, monocyte-to-lymphocyte ratio; Neu, neutrophil; NLR, neutrophil-to-lymphocyte ratio; PLR, platelet-to-lymphocyte ratio; SII, systemic immune-inflammation index.

**Table 3.** The Median Levels of Serum HGF in Relation to Laboratory Parameters of the Patients

Characteristics	HGF (ng/mL), Median	Range	*P*
Ca 19-9 (U/mL)			.259
≤62.4	239.72	4.26-1051.06	
>62.4	371.63	1.42-1002.84	
Neutrophil (/μL)			.184
≤4900/μL	229.79	1.42-964.54	
>4900/μL	350.36	49.65-1051.06	
Lymphocyte			.373
≤2100/μL	350.36	1.42-1051.06	
>2100/μL	209.93	5.67-981.56	
Monocyte			**.016**
≤700/μL	218.44	1.42-964.54	
>700/μL	398.58	58.16-1051.06	
Platelet (/mL)			**.018**
≤259 000	229.79	1.42-858.16	
>259 000	407.09	4.26-1051.06	
NLR			.063
≤2.5	209.93	4.26-981.56	
>2.5	370.92	1.42-1051.06	
MLR			.278
≤0.32	234.05	4.26-981.56	
>0.32	357.45	1.42-1051.06	
PLR			.055
≤128.9	219.86	1.42-981.56	
>128.9	398.58	4.26-1051.06	
SII			**.028**
≤613 333.3	224.12	1.42-612.77	
>613 333.3	398.58	4.26-1051.06	
CRP			**.010**
≤9.9 mg/L	176.59	1.42-778.72	
>9.9 mg/L	402.84	5.67-1051.06	
CAR			**.040**
≤2.8	178.72	1.42-858.16	
>2.8	371.63	5.67-1051.06	

Ca 19-9, carbohydrate antigen 19-9; CAR, CRP-to-albumin ratio; CRP, C-reactive protein; HGF, hepatocyte growth factor; MLR, monocyte-to-lymphocyte ratio; NLR, neutrophil-to-lymphocyte ratio; PLR, platelet-to-lymphocyte ratio; SII, systemic immune-inflammation index.

**Table 4.** Univariate survival Analysis (Kaplan–Meier) and Log-Rank Test

Characteristics	Median Overall Survival (Months)	95% CI	Log-Rank *P*
Age			**.007**
≤65	20.9	10.214-31.586	
>65	4.5	2.956-6.111	
Gender			.534
Male	12.7	6.668-18.665	
Female	14.2	8.684-19.782	
Tumor type			.111
Pancreatic cancer	12.1	3.822-20.445	
Biliary tract cancer	12.8	5.134-20.533	
Ampullary cancer	32.4	NA	
Metastasis			**.008**
M0	18.3	9.416-27.184	
M1	5.0	0.668-9.399	
Ca 19-9			**.016**
≤62.4 U/mL	21.6	13.403-29.864	
>62.4 U/mL	7.8	1.906-13.627	
Neutrophil			.404
≤4900 /μL	14.2	4.671-23.795	
>4900 /μL	10.4	2.502-18.232	
Lymphocyte			.740
≤2100 /μL	12.1	5.032-19.168	
>2100 /μL	15.5	6.793-24.207	
Monocyte			.339
≤700 /μL	20.5	10.131-30.869	
>700 /μL	12.1	0.342-23.924	
NLR			.445
≤2.5	13.2	9.495-16.839	
>2.5	10.4	0.000-29.294	
MLR			**.010**
≤0.32	20.9	14.325-27.475	
>0.32	7.7	1.873-13.594	
PLR			.509
≤128.9	13.2	10.304-16.029	
>128.9	12.8	0.00-32.641	
SII			.859
≤613 333.3	13.2	11.1-15.3	
>613 333.3	12.1	0.0-39.3	
CRP			.381
≤9.9 mg/L	12.8	1.2-24.4	
>9.9 mg/L	10.4	1.6-19.1	
CAR			.841
≤2.8	12.8	0.0-25.9	
>2.8	13.2	5.9-20.5	
HGF (ng/mL)			.819
≤329.1	13.2	0.078-26.255	
>329.1	12.8	6.907-18.760	

Ca 19-9, carbohydrate antigen 19-9; CAR, CRP-to-albumin ratio; CRP, C-reactive protein; HGF, hepatocyte growth factor; MLR, monocyte-to-lymphocyte ratio; NLR, neutrophil-to-lymphocyte ratio; PLR, platelet-to-lymphocyte ratio; SII, systemic immune-inflammation index.
